# Comparative Analysis of Morphometric, Densitometric, and Mechanical Properties of Skeletal Locomotor Elements in Three Duck Species (*Anatidae: Anatinae*)

**DOI:** 10.3390/ani14152191

**Published:** 2024-07-27

**Authors:** Cezary Osiak-Wicha, Ewa Tomaszewska, Siemowit Muszyński, Marian Flis, Michał Świetlicki, Marcin B. Arciszewski

**Affiliations:** 1 Department of Animal Anatomy and Histology, Faculty of Veterinary Medicine, University of Life Sciences in Lublin, Akademicka 12, 20-950 Lublin, Poland; cezary.wicha@up.lublin.pl; 2 Department of Animal Physiology, Faculty of Veterinary Medicine, University of Life Sciences in Lublin, Akademicka 12, 20-950 Lublin, Poland; ewarst@interia.pl; 3 Department of Biophysics, Faculty of Environmental Biology, University of Life Sciences in Lublin, Akademicka 13, 20-950 Lublin, Poland; siemowit.muszynski@up.lublin.pl; 4 Department of Ethology and Wildlife Management, Faculty of Animal Sciences and Bioeconomy, University of Life Sciences in Lublin, Akademicka 13, 20-950 Lublin, Poland; marian.flis@up.lublin.pl; 5 Department of Applied Physics, Faculty of Mechanical Engineering, Lublin University of Technology, Nadbystrzycka 36, 20-618 Lublin, Poland; m.swietlicki@pollub.pl

**Keywords:** avian biomechanics, adaptation, flight behaviour, mallard, teal, tufted duck

## Abstract

**Simple Summary:**

This study aims to investigate the skeletal adaptations of three duck species: the Mallard, Tufted Duck, and Green-Winged Teal. Ducks play a critical role in wetland ecosystems by aiding in seed dispersal and nutrient cycling. To understand how their skeletal structures support different survival strategies and modes of locomotion, this research focuses on the tibiotarsus and humerus bones. Bone samples were collected from deceased ducks, cleaned, and measured for length, weight, and density. Using dual-energy X-ray absorptiometry, bone mineral density (BMD) and content (BMC) were quantified, while mechanical properties such as yield force and stiffness were assessed through a 3-point bending test. The findings demonstrate that each species exhibits unique bone characteristics suited to their specific behaviours and habitats. Mallards, known for their versatility, possess stronger and denser bones, which are advantageous for various environments. They show the highest Seedor index, indicating robust bone structure. Teals, being smaller and capable of rapid flight, have lighter and less dense bones, which are beneficial for quick movements in shallow wetlands but exhibit lower BMD and BMC. Tufted Ducks, which are adapted for deep diving, have bones that are particularly strong and stiff, allowing them to forage effectively underwater. These variations in bone structure and density suggest adaptations to each species’ specific ecological strategies and survival mechanisms, reflecting their ecological roles and survival strategies. Understanding these adaptations may provide valuable insights into the functional morphology of ducks.

**Abstract:**

Ducks (*Anatinae*) play a crucial role in wetland ecosystems, contributing to seed dispersal and nutrient cycling. This study investigates the skeletal adaptations of three duck species: the Mallard (*Anas platyrhynchos*), Tufted Duck (*Aythya fuligula*), and Green-Winged Teal (*Anas crecca*). The focus is on the tibiotarsus and humerus bones to understand how these adaptations support their different locomotion and habitat preferences. Bone samples n = 6 of deceased ducks (both male and female) from each species (for a total of 36 samples) were cleaned and measured for length, weight, and density. Dual-energy X-ray absorptiometry was used to determine bone mineral density (BMD) and bone mineral content (BMC), and mechanical properties like yield force and stiffness were tested using a 3-point bending test. The results show significant differences in body weight, bone weight, and bone length among the species, with Mallards being the largest and Teals the smallest. Male Teals displayed higher relative bone weight (RBW) in their tibia compared to male Mallards, and male Mallards had significantly lower RBW in the humerus compared to the other species. Female Teals had higher RBW than the other species. Teals also exhibited much lower BMD in the tibia, whereas female Mallards had lower BMD in the humerus. The Seedor index revealed that male Mallards had the highest values in the tibia, while female Teals had the lowest. Mechanical testing indicated that Teals had lower yield force and breaking force in the tibia, whereas Mallards showed the highest stiffness in both bones. Tufted Ducks had intermediate values, consistent with their diving behaviour. These findings suggest that the Mallard’s robust bones support its adaptability to various environments and diverse locomotion and foraging strategies. The Teal’s lighter and less dense bones facilitate rapid flight and agility in shallow wetlands. The Tufted Duck’s intermediate bone characteristics reflect its specialization in diving, requiring a balance of strength and flexibility. Understanding these skeletal differences may provide valuable insights into the evolutionary biology and biomechanics of these species, aiding in their conservation and enhancing our knowledge of their roles in wetland ecosystems. By exploring the functional morphology of these ducks, this study aims to shed light on the biomechanical mechanisms that underpin their locomotion and foraging behaviours.

## 1. Introduction

Ducks (*Anatinae*) are a diverse and ecologically significant group of waterfowl distributed across various habitats worldwide [1]. Characterized by their adaptability and migratory behaviours, ducks play crucial roles in ecosystem dynamics, contributing to seed dispersal, nutrient cycling, and maintaining wetland biodiversity [2].

Among the numerous duck species, the Polish avifauna boasts notable diversity, inhabiting wetlands, lakes, rivers, and coastal regions. Notable among these are the Mallard (*Anas platyrhynchos*), Tufted Duck (*Aythya fuligula*), and Green-Winged Teal (hereafter referred to as Teal) (*Anas crecca*), collectively representing distinct ecological adaptations, diet, behavioural patterns, and physiological adaptations [1,3]. The Mallard, recognized for its iridescent green head and distinctive quacking call, is renowned for its omnivorous diet and adaptable nature, often observed in diverse aquatic environments [4,5,6]. In contrast, the Tufted Duck, characterized by its sleek black-and-white plumage and distinctive tuft of feathers at the back of its head, predominantly feeds on aquatic vegetation and invertebrates, diving to considerable depths in pursuit of sustenance [7,8]. The Teal, renowned for its small size and rapid flight, possesses a more insectivorous diet and is frequently observed in shallow wetlands and marshes [5,9].

The differentiation in flight patterns, feeding behaviours, and habitat preferences among these species underscores the necessity for diverse skeletal adaptations to support their locomotion, foraging, and survival strategies [10,11]. The tibiotarsus (hereafter referred to as tibia) and humerus, as pivotal skeletal elements, play crucial roles in avian locomotion and energy expenditure. The tibia provides structural support and facilitates propulsion during walking, anchoring leg musculature and aiding terrestrial mobility and take-off during flight. Its resistance to impact and role in the walking cycle are essential for understanding avian locomotion. In contrast, the femur plays a more limited role in propulsion and is primarily involved in the attachment of muscles for leg movement. The tibia’s morphology is thus more directly correlated with its function in supporting and propelling birds during terrestrial locomotion, making it a critical bone for these activities, facilitating terrestrial mobility and take-off during flight [12]. In comparison, the humerus serves as the primary lever for wing propulsion, enabling sustained flight and aerial maneuverability [13,14,15]. Avian bones exhibit differences from mammalian bones, particularly in pneumatization and density. Pneumatized bones have lower cortical thickness and bending strength. Avian bones, such as the cranium, humerus, and femur, show higher density compared to bats and terrestrial mammals, suggesting adaptations for maximizing strength and stiffness while minimizing mass. These insights highlight the importance of bone morphology, density, and mechanical properties in flight evolution [16,17]. Understanding the morphometric, densitometric, and mechanical properties of these skeletal elements is paramount for elucidating the biomechanical mechanisms underpinning avian locomotion and foraging behaviours. Moreover, unravelling interspecies variations in bone architecture and strength has profound implications for evolutionary biology, biomechanics, and conservation science.

In this study, we conduct a comprehensive comparative analysis of the morphometric, densitometric, and mechanical properties of the tibia and humerus across three duck species: the Tufted Duck, Mallard, and Teal. We chose the tibia due to its significant involvement in locomotion and observed adaptive changes in bone mass and mineralization during periods of increased muscle attachment. Conversely, we did not select the femur for examination as it may not undergo the same changes as the tibia during moult periods. The tibia shows significant changes in mass and mineralization, likely due to increased demands for strength and muscle attachment during terrestrial locomotion, which are not as pronounced in the femur. Studies have shown that during the moult period in captive Barnacle Geese, the femur exhibits a decrease in mineral content, suggesting it plays a different role in adaptive remodelling compared to the tibia, which is more directly involved in support and propulsion during walking [18]. Additionally, we investigate the ratio of sternal crest height to sternum length (Ch/Sl ratio). The sternal crest, a prominent feature of the sternum, serves as an attachment site for flight muscles and plays a crucial role in supporting the pectoral muscles during wing beats. The size of the sternal crest in birds shows correlations with body size and weight. Research indicates that sternal morphology is linked to locomotory capabilities, with sternums of flying birds having equal width and height, whereas swimming birds exhibit greater width than height, and walking birds show greater height than width [19,20].

Our hypothesis posits that there will be significant interspecies variation in the different bone properties of the tibia and humerus among the three duck species. Specifically, we anticipate that these variations will correlate with each species’ unique locomotor behaviours, flight patterns, foraging strategies, and habitat preferences. We hypothesize that species engaging in longer migratory flights will exhibit tibiae and humeri with higher mechanical strength and optimized mineral density to support sustained endurance, whereas species that navigate dense wetland vegetation will show adaptations such as denser bone structures to withstand rapid, agile movements. By testing these hypotheses, we aim to elucidate the skeletal adaptations that underpin avian locomotion in diverse ecological contexts, thereby contributing to a deeper understanding of the functional morphology of duck species and their role in wetland ecosystems.

## 2. Materials and Methods

### 2.1. Animals and Research Material

**Figure 1:**
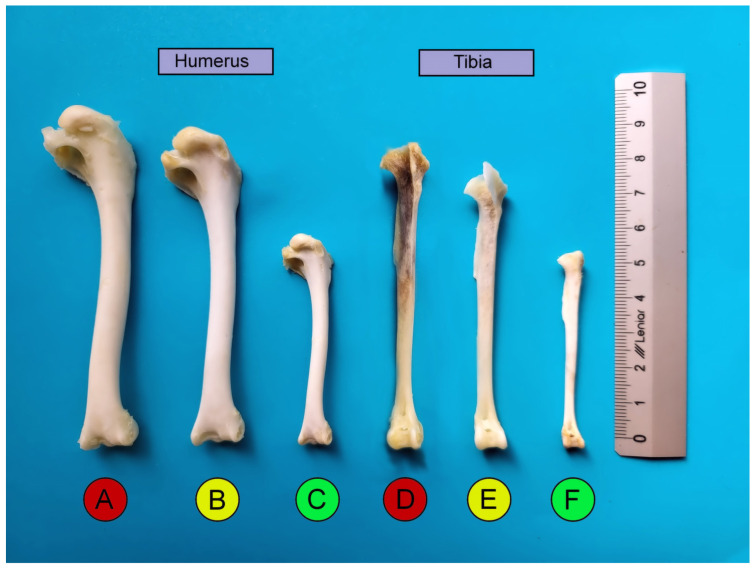
Comparative morphology of the long bones between the three duck species: (**A**) Mallard humerus, (**B**) Tufted Duck humerus, (**C**) Teal humerus, (**D**) Mallard tibia, (**E**) Tufted Duck tibia, (**F**) Teal tibia.

The research material comprised deceased ducks from the Tufted Duck, Mallard and Teal species harvested during the hunting season in July 2023, in accordance with the regulations outlined in the Polish hunting law (Act–Law on hunting 1995; https://isap.sejm.gov.pl/isap.nsf/DocDetails.xsp?id=wdu19951470713; accessed on 26 July 2004). Ethical approval was not required for this study, as bone samples were obtained post-mortem from deceased animals. Each animal’s weight was recorded, and its sex was determined by an examination of the gonads. Following careful dissection, the humerus, tibia, and sternum were meticulously cleaned of any adhering tissues using surgical scissors and a scalpel. Subsequently, these bones were measured for length and weight. Samples were categorized into groups based on sex and species. The first n = 6 shot individuals for each species and sex were used for the study, for a total of 36 specimens. Relative bone weight (RBW) and the Seedor index, serving as an indicator of whole bone density, were calculated from the measurements obtained for the tibia and humerus (Figure 1). RBW was determined by calculating the ratio of bone weight to the total body weight of the respective animal. The Seedor index was calculated as the ratio of bone weight to bone length. Following measurement and calculation procedures, the bones were individually placed in sealed ziplock bags to ensure preservation. Subsequently, the bones were stored at −20 °C in a freezer until required for bone densitometry analyses and mechanical testing at a later stage of the study.

### 2.2. Dual-Energy X-ray Absorptiometry

Bone mineral density (BMD) and bone mineral content (BMC) were measured using dual-energy X-ray absorptiometry (DXA) on a Lunar iDXA densitometer (GE, Madison, WI, USA). The instrument was calibrated with densitometer-specific phantoms according to the manufacturer’s guidelines to ensure accuracy within the BMD range of 0.6–1.4 g/cm^2^. The scanning was carried out in “small animal” mode with the enCORE software version 17.0 (GE, Madison, WI, USA). Bone specimens were placed on a specialized pad designed for small animal scans, provided by the densitometer manufacturer. BMD and BMC measurements were obtained from the data by defining regions of interest that included the entire bone structure. To maintain consistency and reliability, all measurements were conducted by the same trained technician.

### 2.3. Bone Analysis

To evaluate the mechanical properties of the mid-diaphysis bones, a 3-point bending test was performed using a Zwick Z010 universal testing machine (Zwick, Ulm, Germany). The bones were subjected to a loading rate of 10 mm/min until fracture occurred. Load–deflection curves were recorded to determine the various mechanical properties of the tibia and humerus, including yield force (F_yield_), elastic work (W_yield_), stiffness within the elastic deformation region, breaking force (F_max_), and breaking work (W_max_) at the point of fracture. After the mechanical tests, the bones were cut at the midpoint of the diaphysis using a diamond bandsaw (MBS 240/E, Proxxon GmbH, Foehren, Germany). Subsequently, the external transversal (H_out_) and internal transversal (H_inn_), as well as external anteroposterior (V_out_) and internal anterioposterior (V_inn_) diameters of the mid-diaphysis cross-section were measured using a digital calliper. These measurements facilitated the determination of the mid-diaphysis geometric parameters, including mean relative wall thickness (MRWT), cortical index (CI), cross-sectional area (CSA), and cross-sectional moment of inertia (Ix). Utilizing the previously established mechanical properties of the tibia and humerus, in combination with the calculated mid-diaphysis geometrical parameters, femur material properties were derived. These properties encompassed yield strain (Ɛ_yield_), yield stress (σ_yield_), breaking strain (Ɛ_max_), and breaking stress (ơ_max_) [21]. The details of all calculations with formulas can be found in previous works [13].

### 2.4. Statistical Analysis

Mean values and standard errors (SE) were utilized to present all findings. The normal distribution of variables was assessed using the Shapiro–Wilk test of normality, whereas the equality of variance was verified through Levene’s test. For normally distributed data, a two-way Analysis of Variance (ANOVA) followed by Tukey’s Honest Significant Difference was employed. Two factors were taken into consideration, sex and species. In cases where the data did not exhibit normal distribution, multiple Kruskal–Wallis tests were conducted. Comparisons were made based on sex and species. Statistical significance was determined at *p* < 0.05. Analysis was performed using GraphPad Prism ver. 9.5.1 for Windows (GraphPad Software, San Diego, CA, USA).

## 3. Results

### 3.1. Physical Characteristics

**Figure 2:**
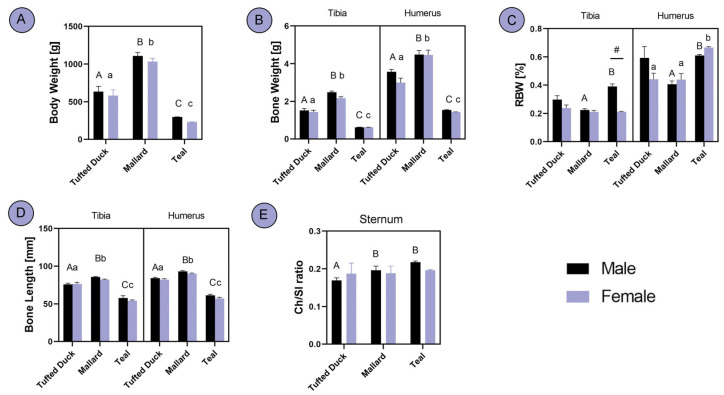
Analysis of body dimensions between the Tufted Duck, Mallard and Teal duck species males and females: (**A**) body weight, (**B**) bone weight, (**C**) relative bone weight, (**D**) bone length, (**E**) sternal crest high to sternum length ratio. The letters indicate significant differences between species, with uppercase letters denoting distinctions among males and lowercase letters representing variations among females. Statistical significance was determined at *p* < 0.05.

Significant differences in body weight, bone weight, and bone length, of both bones, were observed among all the three studied duck species, with the Mallard being the biggest and Teal the smallest in size (Figure 2A, Figure 2B, Figure 2D, respectively; Figure 1; *p* < 0.001). However, regarding the RBW of the tibia, a significant difference was observed between male Mallards and male Teals (*p* < 0.05), with the latter demonstrating higher RBW. In the humerus, male Mallards exhibited significantly lower RBW compared to the other two species (*p* < 0.01), whereas female Teals displayed higher RBW than the other two species (*p* < 0.001). Additionally, a sex-dependent difference was noted in the RBW of Teal tibia, with males showing significantly higher RBW (Figure 2C; *p* < 0.05). In the sternal crest height to sternum length ratio, the differences were noticed only between males, with Tufted Ducks having a lower ratio then Mallards (*p* < 0.05) and Teals (Figure 2E; *p* < 0.001). No significant differences were noted between sexes within each species.

### 3.2. Bone Properties

**Figure 3:**
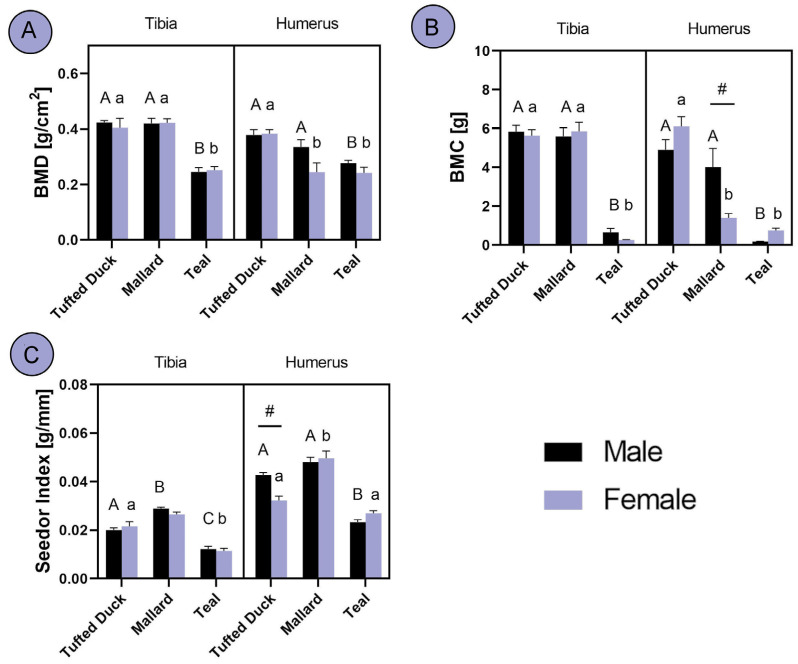
Analysis of differences in tibia and humerus properties between the Tufted Duck, Mallard and Teal duck species males and females: (**A**) bone mineral density, (**B**) bone mineral content (**C**) Seedor index. The letters indicate significant differences between species, with uppercase letters denoting distinctions among males and lowercase letters representing variations among females. Hash (#) indicates significant differences between sexes within one species. Statistical significance was determined at *p* < 0.05.

An analysis of the BMD revealed significant differences, with Teals displaying much lower BMD in the tibia compared to the other two species (*p* < 0.001). In humerus, BMD was similar to tibia; however, female Mallards exhibited significantly lower BMD and were closer to Teals’ levels (Figure 3A; *p* < 0.001). In terms of BMC, Teals had significantly lower levels in the tibia compared to the other two species (*p* < 0.001), whereas in the humerus, BMC, similarly to BMD, was lower in female Mallards and closer to Teals’ levels (Figure 3B; *p* < 0.001). An evaluation of the Seedor index in the tibia revealed significant differences among all male ducks, with Mallards having the highest index (*p* < 0.01) and Teals the lowest (*p* < 0.05). Among females, Tufted Ducks and Mallards exhibited similar Seedor indexes, whereas female Teals displayed the lowest index (*p* < 0.01). In the humerus, male Teals exhibited a lower Seedor index compared to the other two species, whereas female Mallards displayed a higher index than the other two species (*p* < 0.01). Additionally, a sex-based difference in Tufted Ducks was noted, with males having a higher Seedor index than females (Figure 3C; *p* < 0.001).

### 3.3. Geometrical Properties

**Figure 4:**
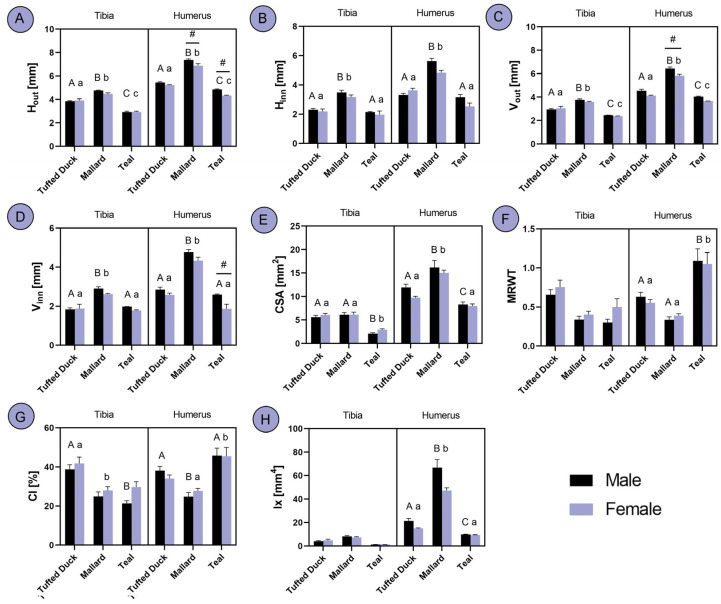
Analysis of differences in tibia and humerus geometrical properties between the Tufted Duck, Mallard and Teal duck species males and females: (**A**) transversal outer diameter, (**B**) transversal inner diameter, (**C**) anteroposterior outer diameter, (**D**) anteroposterior inner diameter, (**E**) mid-diaphysis cross-sectional area, (**F**) mean relative wall thickness, (**G**) cortical index, (**H**) cross-sectional moment of inertia. The letters indicate significant differences between species, with uppercase letters denoting distinctions among males and lowercase letters representing variations among females. Hash (#) indicates significant differences between sexes within one species. Statistical significance was determined at *p* < 0.05.

Significant differences in Hout were observed in both the tibia and humerus across all studied duck species, with Mallards exhibiting the highest values and Teals the lowest (*p* < 0.001). Sexual dimorphism was evident in Mallards and Teals, where males demonstrated higher Hout values compared to females (Figure 4A; *p* < 0.01). In contrast, differences in H_inn_ were consistent between the tibia and humerus, with no significant variation observed between Teals and Tufted Ducks, whereas Mallards displayed higher values than both (Figure 4B; *p* < 0.001). An analysis of V_out_ revealed significant differences among all three species in both the tibia and humerus (*p* < 0.001). Notably, in the humerus of Mallards, a sex-dependent difference was noted, with males displaying higher V_out_ values (Figure 4C; *p* < 0.001). Similarly, in V_inn_, Tufted Ducks and Teals exhibited similar values in both the tibia and humerus (*p* < 0.001), whereas Mallards displayed higher values than both species (*p* < 0.01) Additionally, sexual dimorphism was observed in the Teal humerus, with males demonstrating higher V_inn_ values (Figure 4D; *p* < 0.05). Regarding the CSA of the tibia, Tufted Ducks and Mallards exhibited similar values (*p* < 0.01), whereas Teals displayed a lower CSA (*p* < 0.05). In the humerus, all three species differed significantly among males, with Mallards displaying the highest CSA and Teals the lowest (*p* < 0.001). However, among females, Tufted Ducks and Teals exhibited similar CSA values (*p* < 0.05), whereas Mallards displayed a higher CSA (Figure 4E; *p* < 0.001). No significant differences were observed in MRWT in the tibia, whereas in the humerus, Teals displayed significantly higher MRWT values than Mallards (*p* < 0.001) and Tufted Ducks (Figure 4F; *p* < 0.05). In terms of CI, Tufted Ducks exhibited higher values than Mallards (*p* < 0.05) and Teals (*p* < 0.01) in the tibia, whereas in the humerus, Mallards displayed lower CI values than the other two species (Figure 4G; *p* < 0.05). No significant differences were observed in Ix in the tibia (*p* > 0.05). In the male humerus, all three species differed, with Mallards having significantly higher Ix then Teal (*p* < 0.001) and Tufted Ducks (*p* < 0.05), whereas in females, Tufted Ducks and Teals exhibited similar values, with Mallards displaying significantly higher Ix (Figure 4H; *p* < 0.001).

### 3.4. Mechanical Properties

**Figure 5:**
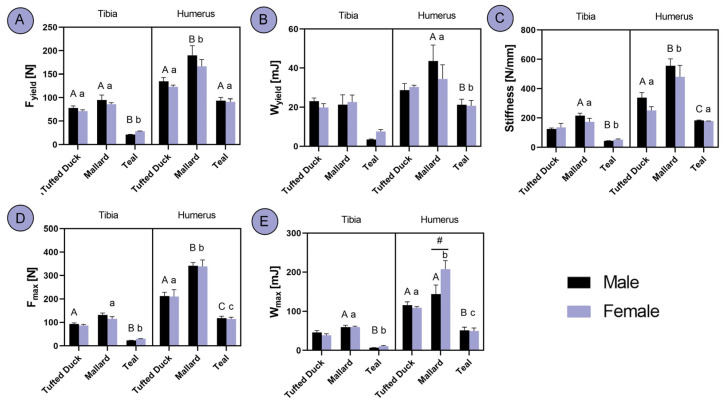
Analysis of differences in tibia and humerus mechanical properties between the Tufted Duck, Mallard and Teal duck species males and females: (**A**) yield force, (**B**) elastic work, (**C**) stiffness, (**D**) breaking force, (**E**) breaking work. The letters indicate significant differences between species, with uppercase letters denoting distinctions among males and lowercase letters representing variations among females. Hash (#) indicates significant differences between sexes within one species. Statistical significance was determined at *p* < 0.05.

Significant differences in F_yield_ were observed among the three studied duck species in the tibia, with Teals exhibiting significantly lower values compared to the other two species (*p* < 0.001). In the humerus, Tufted Ducks and Teals showed similar F_yield_ values, whereas Mallards displayed higher values (Figure 5A; *p* < 0.001). Regarding W_yield_, no significant differences were observed in the tibia, whereas in the humerus, Tufted Ducks and Teals exhibited similar W_yield_ values, with Mallards displaying higher values (Figure 5B; *p* < 0.001). In terms of stiffness, Mallards demonstrated higher levels than the other two species in the tibia (*p* < 0.05). In the humerus, all three species differed significantly among males, with Mallards exhibiting the highest stiffness and Teals the lowest (*p* < 0.01). Among females, Tufted Ducks and Teals showed similar stiffness values, whereas Mallards displayed higher levels (Figure 5C; *p* < 0.001). An analysis of F_max_ revealed that Teals had lower values compared to the other two species in the tibia (*p* < 0.05). In the humerus, all three species differed significantly, with Mallards exhibiting the highest F_max_ and Teals the lowest (Figure 5D; *p* < 0.001). In terms of W_max_ in the tibia, Tufted Ducks and Mallards exhibited similar values among males, whereas Teals had lower values (*p* < 0.05). Among females, all three species differed significantly, with Mallards displaying the highest values and Teals the lowest (*p* < 0.001). Additionally, a sex-based difference was observed in Mallards, with females exhibiting significantly higher W_max_ (Figure 5E; *p* < 0.001).

### 3.5. Bone Material Properties

**Figure 6:**
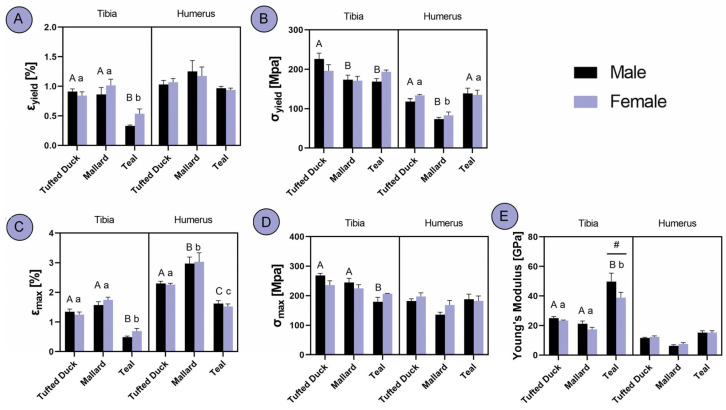
Analysis of differences in tibia and humerus bone material properties between the Tufted Duck, Mallard and Teal duck species males and females: (**A**) yield strain, (**B**) yield stress, (**C**) breaking strain, (**D**) breaking stress, (**E**) Young’s modulus. The letters indicate significant differences between species, with uppercase letters denoting distinctions among males and lowercase letters representing variations among females. Hash (#) indicates significant differences between sexes within one species. Statistical significance was determined at *p* < 0.05.

Significant differences were observed in ɛ_yield_ in the tibia among the studied duck species, with Teals exhibiting significantly lower values compared to the other two species (*p* < 0.001). No differences were found in the humerus (Figure 6A). In terms of σ_yield_, differences were observed only among males in the tibia, where Tufted Ducks displayed higher values compared to the other two species (*p* < 0.05). In the humerus, Tufted Ducks and Teals showed similar σ_yield_ values, whereas Mallards exhibited lower values (Figure 6B; *p* < 0.001). An analysis of ɛ_max_ revealed that Tufted Ducks and Mallards displayed similar values in the tibia, whereas Teals exhibited lower values (*p* < 0.001). In the humerus, all three species differed significantly, with Mallards exhibiting the highest ɛ_max_ (*p* < 0.01) and Teals the lowest (Figure 6C; *p* < 0.001). Regarding σ_max_ in the tibia, differences were observed only among males, with Teals displaying lower values compared to the other two species (*p* < 0.01). No differences were observed in the humerus (Figure 6D). In terms of Young’s modulus, Tufted Ducks and Mallards exhibited similar values in tibia, whereas Teals displayed significantly higher modulus values (*p* < 0.001). Additionally, a sex-based difference was observed in Teals, with males exhibiting higher values (*p* < 0.01). No differences were observed in the humerus (Figure 6E).

## 4. Discussion

In contrast to other terrestrial vertebrates using bipedal locomotion, birds possess two distinct locomotor systems. Their forelimbs, or wings, primarily serve for aerial locomotion, whereas their hindlimbs, or legs, are utilized for terrestrial locomotion and water locomotion, such as swimming. Notably, the major flight muscles, including the pectoralis and supracoracoideus, attach to the keel bone, facilitating flapping flight by contracting and exerting forces on the keel bone. The resistance encountered during physical exercise promotes muscle hypertrophy, whereas a lack of exercise induces muscle atrophy. This phenomenon is exemplified in waterfowl during moult, where the loss of flight feathers renders them temporarily flightless, prompting a transition to terrestrial locomotion and consequent changes in body weight, flight muscle atrophy, and hindlimb hypertrophy [22,23]. In birds, pneumaticity, or the presence of air-filled cavities within bones, varies across different skeletal elements. Whereas pneumaticity is commonly observed in bones such as the humerus and tibia, it affects their mechanical properties differently. Bones with pneumaticity tend to be lighter in weight due to the presence of air sacs, which influences their overall strength and stiffness. For example, the humerus, being pneumatic and subjected to torsional forces during flight, may exhibit greater flexibility and lower density. The tibia, also pneumatic but primarily supporting terrestrial locomotion, experiences compressive forces and must balance lightness with the need for strength and stability. This dual requirement influences the tibia’s mechanical properties, such as strength, elasticity, and resistance to stress, ensuring it can support the bird during walking, running, and swimming [24].

The observed differences in body size, bone dimensions, and bone weight reflect each species’ distinct flight behaviours and physical adaptations. The Mallard, the largest of the three ducks, displayed the most robust skeletal elements, likely reflecting its need for powerful take-offs for flight and its ability to navigate diverse aquatic environments [25,26,27]. Mallards can also initiate rapid take-offs when threatened, showcasing their versatile escape capabilities. Conversely, the Teal, the smallest species, exhibited the most lightweight bones, which likely facilitate its maneuverability and rapid escape flights. While Teals excel in quick take-offs, the Tufted Duck requires a longer run-up for take-off, characteristic of its diving behaviour. This distinction underscores the differential locomotor strategies between dabbling ducks (tribe *Anatini*), such as Mallards and Teals, known for their rapid take-off abilities, and diving ducks (tribe *Aythini*), like Tufted Ducks, which necessitate an extended run-up. Previous studies have supported correlations between skeletal morphology and locomotor functions in birds, highlighting how bone dimensions align with flight and swimming capabilities [19]. Skeletal adaptations in aquatic birds include changes in bone microstructure, such as differences in compactness and remodelling [27]. The appendicular skeletons of diving birds show specific features that aid in underwater locomotion, with variations in bone structure distinguishing divers from surface swimmers and flyers. Furthermore, the hindlimb muscles of foot-propelled swimming birds, combined with their webbed feet, have evolved to suit the challenges of producing propulsive forces underwater [28,29]. The presence of webbed feet is crucial for efficient swimming, providing a larger surface area for propulsion and contributing to the overall agility and maneuverability in water. These webbed feet, along with robust and well-adapted leg bones, help species like the Tufted Duck to dive and forage effectively underwater. Our findings on the variations in bone dimensions, such as length and width, suggest adaptations for different flight behaviours and physical demands. Ducks with higher RBW or Seedor index, like Mallards, may have evolved adaptations for sustained flight over long distances, engaging in migratory behaviours and navigating varied aquatic environments. Conversely, species with lower RBW or Seedor index, like Teals, may have developed adaptations for rapid flight and maneuverability in confined spaces, such as marshes and wetlands. This suggests that bone density and mass, standardized to the bird’s mass and mass/length ratio, may play a crucial role in determining flight capabilities and locomotor strategies among different duck species. The concept of mosaic evolution, as highlighted by Navalón et al. [30], suggests that different skeletal elements can exhibit distinct evolutionary patterns, with generalist species evolving a more generalized skeletal morphology to accommodate a wider range of locomotor demands. This aligns with our observations of skeletal variations among the studied species.

Interspecies variation was also evident in the bone strength and stiffness. The Mallard exhibited the highest values for yield force, ultimate force, and stiffness in both the tibia and humerus. This suggests that the Mallard’s skeletal elements are optimized to withstand greater mechanical loads during flight and potentially forceful landings. Forceful landings refer to the significant impact forces experienced during touchdown, which in the case of Mallards, involves landing on water. Mallards employ a controlled-collision strategy when landing, which involves maintaining a constant ratio of the distance to the water surface and the rate of change in that distance. This strategy results in a higher approach speed compared to other flyers like pigeons, hummingbirds, and bats, that land on solid substrates. The compliance of water, being less rigid than solid ground, reduces the impact forces that could otherwise cause injury [31]. Therefore, Mallards are able to land more forcefully without sustaining damage, requiring their bones to be robust and strong to absorb these higher forces effectively. This higher mechanical load during landing is reflected in their bone structure, which shows enhanced strength and stiffness to support their unique landing dynamics.

Studies on the flight patterns of Teals and Mallards reveal interesting insights. Mallards exhibit highly predictable movement behaviour, with regular flights between roosts and foraging sites, influenced by water availability and temperature [32]. When landing, Mallards employ a controlled-collision strategy, regulating their approach by keeping a constant distance to the landing surface [31]. In contrast, the Teal displayed the lowest values for these properties, potentially reflecting a trade-off between bone strength and reduced weight for enhanced maneuverability, which is supported by research on avian skeletal pneumaticity [33]. Studies have shown that Teal species exhibit adaptations in their bones related to migration and flight patterns, such as longer coracoids and sternums, favouring flight ability [34]. Additionally, research on the Blue-winged Teal (*Spatula discors*) indicates a reduced adrenocortical response during migration, potentially protecting flight muscles from stress [35]. Furthermore, investigations on speckled Teal populations revealed genetic patterns associated with high-altitude adaptation, including loci involved in bone morphogenesis and oxidative phosphorylation, potentially contributing to flight adaptations [36]. Interestingly, Tufted Ducks displayed intermediate values for most mechanical properties, potentially reflecting their adaptation for diving and underwater locomotion, requiring a balance between bone strength and streamlining [37].

The observed variations in bone material properties among duck species support their functional specializations [38,39,40]. For instance, the Teal exhibited lower yield strain and ultimate strain compared to other ducks, suggesting less elastic bone material. This characteristic may be an adaptation for rapid wing beats during agile flight maneuvers but could potentially increase susceptibility to microdamage accumulation [41]. Additionally, the histomorphometry analysis of wing bones in different avian species revealed variations in bone mineral density and morphology, reflecting adaptations to different flight styles. These findings collectively highlight how bone material properties are intricately linked to the specific functional demands of flight in different bird species, showcasing adaptations that optimize performance but may also pose trade-offs in terms of bone resilience [39,42,43]. The higher yield strain in Mallards, coupled with comparable yield stress, suggests a bone structure capable of withstanding greater deformation before failure. This may indicate a bone material optimized for absorbing impact energy and providing resilience during forceful landings, which could be advantageous for Mallards, known for their sustained flight over long distances and varied habitats. Additionally, the higher yield strain could imply a greater ability to adapt to the varying loads and stresses encountered during flight and terrestrial locomotion, contributing to the Mallard’s versatility in different environmental conditions [2,10,31]. Conversely, the lower yield strain in Teals may indicate a bone structure less able to withstand deformation before failure compared to Mallards, despite similar yield stress levels. This could suggest that Teals prioritize other aspects of bone functionality, such as minimizing bone mass to enhance flight maneuverability or optimizing bone strength for rapid take-offs and evasive maneuvers in their habitat. These findings align with the concept of strain adaptation, where bone material properties adjust to the mechanical demands placed upon them [44,45]. The morphological variations observed in our study’s duck species likely extend beyond flight and can be linked to feeding strategies as well. Kooloos and Zweers [46] proposed a deductive methodology to explain the diversity of feeding mechanisms in ducks, and although their research primarily investigates beak modifications, they posit that complementary adaptations in other skeletal structures may also contribute to feeding behaviours.

Our analysis of the Ch/Sl ratio revealed that differences were observed specifically among males across all three studied duck species, whereas no significant distinctions were detected among females within each species. Interestingly, the ratio was notably higher in Mallards and Teals when compared to Tufted Ducks, with Teals exhibiting the highest values. This disparity in the ratio among species may suggest potential variations in flight performance and muscle attachment efficiency. The study by Dietz et al. highlights the critical relationship between muscle mass and flight efficiency, indicating that birds undergo significant adjustments in muscle mass to accommodate changes in body mass during migration [47]. This dynamic interplay between body mass and muscle physiology underscores the importance of skeletal adaptations to support efficient flight performance. In light of this, the observed differences in the Ch/Sl ratio among duck species take on added significance. The higher Ch/Sl ratio in Mallards and Teals, particularly pronounced in Teals, suggests a potentially greater surface area for muscle attachment, which could facilitate more robust wing propulsion and superior flight performance compared to Tufted Ducks [13,19,48,49]. This implies potential adaptations honed to suit their respective habitat and flight behaviours. Given the rapid flight capability of Teals and the larger body size of Mallards, the divergent ratios likely signify optimizations for distinct flight strategies and environmental exigencies.

The inter-sexual differences in bone properties among Mallards, Tufted Ducks, and Teals reveal intricate adaptations linked to their distinct ecological roles and reproductive pressures. Although the expected size-related differences are straightforward, deeper insights emerge from variations in bone morphology and mechanics. For example, the higher relative bone weight (RBW) in male Teal tibiae likely enhances leg strength for navigating dense wetlands, whereas the lower RBW in male Mallard humeri may optimize flight agility. Structural differences, such as larger outer diameters and cross-sectional areas in male bones, suggest adaptations for enduring greater mechanical stresses, vital for behaviours like flight and territorial defence. Material properties, like lower bone mineral density (BMD) in female Mallards, reflect a physiological trade-off favouring reproductive calcium demands. Mechanical strength disparities, with males generally exhibiting higher yield and breaking forces, further indicate an evolutionary response to the demands of combat and sustained activity. These findings highlight the complex interplay of ecological and reproductive factors shaping skeletal adaptation in ducks.

## 5. Conclusions

In conclusion, this study highlights the complex relationship between skeletal morphology, mechanical properties, and the ecological adaptations of three duck species. The robust skeletal elements observed in Mallards signify their adaptation for powerful flight and adaptability across diverse environments, including both terrestrial and aquatic habitats. Conversely, the lightweight bones of Teals are indicative of their prioritization of maneuverability, which is crucial for their rapid flight and foraging in shallow wetlands. The intermediate skeletal properties observed in Tufted Ducks suggest potential adaptations for both diving and aerial locomotion, although further research is warranted to elucidate these adaptations fully. Future investigations could delve into the relationship between skeletal morphology and flight kinematics to gain deeper insights into the biomechanical mechanisms underlying avian locomotion.

## Data Availability

The raw data supporting the conclusions of this manuscript will be available upon request.
